# Screening Surface Structure–Electrochemical Activity Relationships of Copper Electrodes under CO_2_ Electroreduction Conditions

**DOI:** 10.1021/acscatal.2c01650

**Published:** 2022-05-19

**Authors:** Oluwasegun J. Wahab, Minkyung Kang, Enrico Daviddi, Marc Walker, Patrick R. Unwin

**Affiliations:** † Department of Chemistry, University of Warwick, Coventry CV4 7AL, U.K.; ‡ Institute for Frontier Materials Deakin University, Burwood, Victoria 3125, Australia; § Department of Physics, University of Warwick, Coventry CV4 7AL, U.K.

**Keywords:** electrochemical reduction, scanning electrochemical cell microscopy (SECCM), copper, carbon dioxide, crystallographic orientation, catalyst structure, single entity electrochemistry

## Abstract

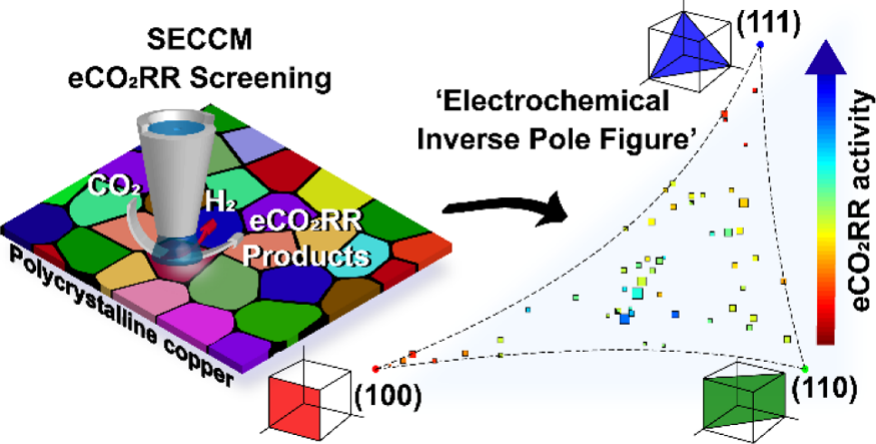

Understanding how crystallographic orientation influences the electrocatalytic performance of metal catalysts can potentially advance the design of catalysts with improved efficiency. Although single crystal electrodes are typically used for such studies, the one-at-a-time preparation procedure limits the range of secondary crystallographic orientations that can be profiled. This work employs scanning electrochemical cell microscopy (SECCM) together with co-located electron backscatter diffraction (EBSD) as a screening technique to investigate how surface crystallographic orientations on polycrystalline copper (Cu) correlate to activity under CO_2_ electroreduction conditions. SECCM measures spatially resolved voltammetry on polycrystalline copper covering low overpotentials of CO_2_ conversion to intermediates, thereby screening the different activity from low-index facets where H_2_ evolution is dominant to high-index facets where more reaction intermediates are expected. This approach allows the acquisition of 2500 voltammograms on approximately 60 different Cu surface facets identified with EBSD. The results show that the order of activity is (111) < (100) < (110) among the Cu primary orientations. The collection of data over a wide range of secondary orientations leads to the construction of an “electrochemical–crystallographic stereographic triangle” that provides a broad comprehension of the trends among Cu secondary surface facets rarely studied in the literature, [particularly (941) and (741)], and clearly shows that the electroreduction activity scales with the step and kink density of these surfaces. This work also reveals that the electrochemical stripping of the passive layer that is naturally formed on Cu in air is strongly grain-dependent, and the relative ease of stripping on low-index facets follows the order of (100) > (111) > (110). This allows a procedure to be implemented, whereby the oxide is removed (to an electrochemically undetectable level) prior to the kinetic analyses of electroreduction activity. SECCM screening allows for the most active surfaces to be ranked and prompts in-depth follow-up studies.

## Introduction

Copper (Cu) is a promising material for electrocatalysis, especially for the electroreduction of carbon dioxide (eCO_2_RR).^1,2^ Cu is the only monometallic catalyst that facilitates CO_2_ conversion to multi-carbon products (i.e., beyond CO and formate).^1,3−7^ This performance is the key to achieving the goal of carbon neutrality and addressing the rising concern of climate change by designing effective catalysts for CO_2_ conversion to useful feedstock.^3,8^ An important characteristic of reactions on Cu electrodes is sensitivity of the reaction kinetics and processes to the surface structure.^1,9,10^

Cu single crystals have been used for eCO_2_RR studies to reveal surface structure–activity relationships and to aid interpretation of results from polycrystalline Cu.^1,11−13^ However, since early eCO_2_RR studies by Hori and colleagues on a broad series of Cu crystals,^1,4,6,14^ experimental and theoretical studies have subsequently focused on a narrower range of crystal orientations and mainly low-index facets.^9,15^ Such streamlining is necessary for in-depth studies, considering that single crystals require multiple steps of surface preparation in one-at-a-time experiments, but limits the range of high-index crystallographic orientations that can be profiled in a reasonable time span. Consequently, the eCO_2_RR activity trends among the numerous high-index Cu crystals that exist on real catalysts remain to be fully explored.

Single crystal studies have produced activity and selectivity trends for Cu grain indexes, albeit with notable disparity.^16−19^ Moreover, recent reports evidence effects of electrode preparation,^19^ pH,^20^ electrolyte species,^21^ and in situ morphological dynamics^22,23^ on eCO_2_RR. Herein, we propose the electrochemical screening of a wide array of Cu surface structures under the same conditions to identify promising structural motifs for further research and eCO_2_RR product analysis.

To rapidly access the electrochemical characteristics of a wide variety of crystallographic sites, we introduced “pseudo-single crystal” electrochemistry,^24^ using high-resolution scanning electrochemical cell microscopy (SECCM)^25−27^ to map out the voltammetry (with several thousand spatially resolved voltammograms easily achievable)^28,29^ on a polycrystalline surface, whose crystallographic structure is determined by co-located electron backscatter diffraction (EBSD). In this way, electrochemical analysis of multiple crystallographic orientations and grain boundaries, found on a polycrystalline metal surface, is achieved^30,31^ as demonstrated by studies of an increasing diversity of systems.^24,28,32−38^ Recent studies have used the combination of SECCM and EBSD to resolve CO_2_ electroreduction activity at grain boundaries of Au,^39−41^ and SECCM alone has been used to study CO_2_ electroreduction at tin/reduced graphene oxide interfaces.^42^

In this work, we employed hopping-mode voltammetric SECCM^28^ to collect spatially resolved linear sweep voltammograms in aqueous KHCO_3_ on polycrystalline copper, under typical eCO_2_RR conditions, with each measurement localized to a ∼ 600 nm-wide spot (defined by the diameter of the SECCM droplet cell). The potential range studied herein is dominated by the H_2_ evolution reaction (HER) on Cu low-index facets^7,19^ but may include eCO_2_RR intermediates on the stepped and kinked high-index facets.^4,19,43^ The original studies of Hori^1^ and later computational studies^44^ propose a correlation between the onset potential at 5 mA cm^–2^ on different Cu surfaces and their performance for producing CH_4_. The current density is in the range of the SECCM studies, so local voltammetric analyses serve as a preliminary indicator of eCO_2_RR activity. As part of our study, we also provide a local electrochemical assay of the naturally formed (upon ambient air exposure) adlayer on different Cu facets. The SECCM configuration is particularly attractive for this analysis as it presents a way to acquire voltammetric measurements immediately after the electrolyte droplet has contacted the Cu surface, which allows analysis of the surface oxide close to the initial state. We then advance protocols to remove the surface oxide at each pixel in the SECCM scan in order to study the electrocatalytic activity at clean Cu surfaces.

The considerable amount of data acquired with SECCM (up to 2500 LSVs over about 60 different grains in a single map) enabled the construction of “electrochemical stereographic triangles”^32^ in which electrocatalytic activity of individual grains is presented with respect to their crystallographic orientations, on a 2D projection which is qualitatively similar to an inverse pole figure (IPF) representation, thereby offering a holistic view of structure–activity that reveals insightful trends across the low-index grains and many high-index crystallographic orientations. This study serves to demonstrate the use of pseudo-single crystal SECCM–EBSD for screening and identifying promising surface features on an electrocatalyst for further investigation.

## Materials and Methods

### Preparation of the Working Electrode and Chemical Reagents

The working electrode/substrate was prepared by embedding a polycrystalline Cu foil (3 mm thickness, size 10 × 10 mm, Goodfellow, U.K., 99.95%) in a circular carbon block with a hot compression mounting machine (SimpliMet, Buehler, USA). The exposed Cu surface was then polished using an AutoMet 300 polishing machine (Buehler, USA), on polishing pads (ChemoMet and TexMat C, Buehler, USA) with 9, 3, and 0.06 μm polishing suspensions (MetaDi Supreme Diamond and MasterMet colloidal silica suspension, Buehler, USA).

In order to remove minor surface deformation after mechanical polishing and to prepare a high-quality flat surface for electrochemical measurements and scanning electron microscopy (SEM), the polycrystalline Cu sample was subjected to vibratory polishing in a non-crystallizing colloidal silica polishing suspension (MasterMet 2, Buehler, USA) at *ca.* 70% vibration amplitude using a VibroMet 2 instrument (Buehler, USA). The polished Cu substrate was then washed with soapy water, followed by rinsing with a copious amount of deionized water and isopropanol to remove the polishing suspension, before being blown dry with argon. The last stage of the sample preparation for the SECCM experiments was surface cleaning with broad Ar^+^ ion beam milling (IM4000Plus, HITACHI, Japan). Between experiments, the polycrystalline Cu substrate was stored in a desiccator equipped with a vacuum pump at room temperature (22 °C).

Potassium chloride (KCl, Honeywell, 99.5%) and potassium bicarbonate (KHCO_3_, Sigma-Aldrich, 99.95%) were used as supplied by the manufacturer. All solutions were prepared with deionized water (ELGA PURELAB systems; resistivity = 18.2 MΩ cm at 25 °C).

### Nanopipettes, Electrolytes, and Quasi-Reference Counter Electrodes

Nanopipettes were fabricated from borosilicate capillaries (GC120F-10, Harvard Apparatus; capillary dimensions: outer diameter, 1.2 mm; inner diameter, 0.69 mm; and length, 100 mm) with a CO_2_ laser puller (Sutter Instruments P-2000). The pulling parameters were as follows: line 1 with HEAT 330, FIL 3, VEL 30, DEL 220, and PUL– and line 2 with HEAT 330, FIL 3, VEL 40, DEL 180, and PUL 120. The nanopipette tip opening was ∼200 nm in diameter.^40^ The nanopipette probe was filled with the 10 mM KHCO_3_ electrolyte, and a silicone oil layer was added on the top of the electrolyte solution in the tip to minimize evaporation from the back during prolonged scanning.^45^ Note that the pH of the electrolyte solution was 5.77 when saturated with CO_2_ and 8.04 when purged with Ar. AgCl-coated Ag wire was used as a quasi-reference counter electrode (QRCE), which was fabricated by electrochemically oxidizing Ag wire (0.125 mm diameter) in saturated KCl solution.^46^ The QRCE was calibrated routinely (before and after the SECCM measurements) in 10 mM KHCO_3_ with respect to a commercial leakless Ag/AgCl electrode (3.4 M KCl, ET072, eDAQ, Australia), resulting in a stable potential of +215 ± 5 mV compared to the standard. Hence, all electrochemical results in this work are presented *versus* Ag/AgCl (3.4 M KCl), referred to as Ag/AgCl. The prepared QRCE was inserted in the back of the nanopipette and positioned ca. 3–4 cm away from the tip end.^46^

### Scanning Electrochemical Cell Microscopy

A home-built SECCM workstation was used for all the SECCM experiments, as detailed in previous studies^29,47,48^ and discussed in brief below. The nanopipette probe was mounted on the z-piezoelectric stage [P-753.2 LISA, Physik Instrumente (PI), Germany] and moved into the initial position using xy-micropositioners (M-461-XYZ-M, Newport, US) and a stepper motor (8303 Picomotor Actuator, Newport, US).

**Figure 1:**
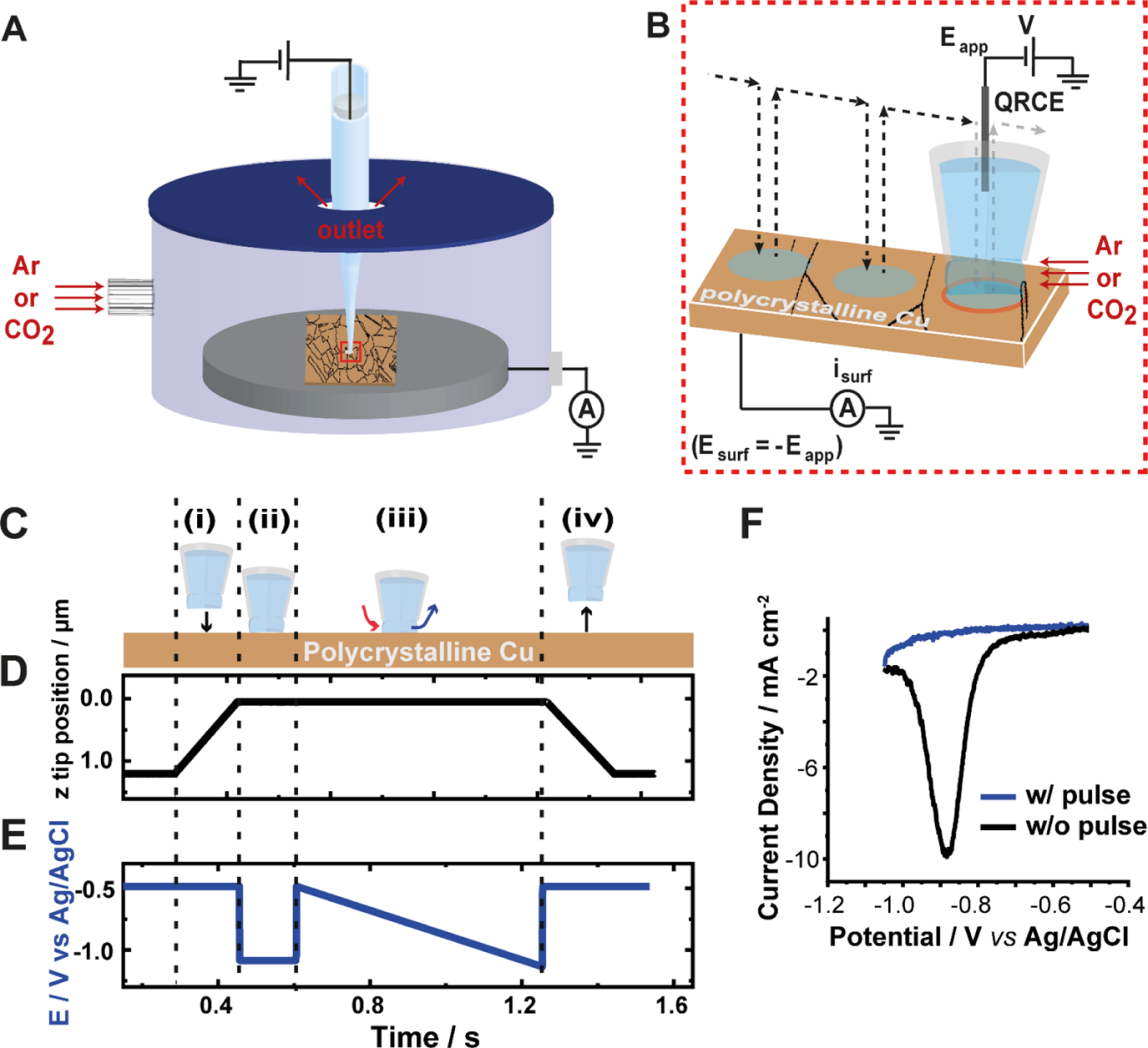
Schematics of the SECCM experimental setup and scanning protocol (not to scale). (A) Nanopipette probe, filled with the 10 mM KHCO_3_ electrolyte and containing a QRCE, in the environmental cell hosting the substrate (polycrystalline Cu embedded in a block of carbon, as schematized through the displayed grain boundaries). Flow of humidified gas (Ar or CO_2_) through the cell, saturating the nanodroplet meniscus and the solution in the lower part of the nanopipette. (B) Expanded view of the nanopipette tip region, illustrating the hopping-mode protocol used in this work. The trajectory of the tip during the scan is shown by the dotted lines with arrowheads, and the area wetted by the nanodroplet (working electrode area) is shown as blue circles. Linear sweep voltammetry (LSV) measurements were made at each hop position by sweeping the potential, *E*_app_, of the QRCE and measuring the current, *i*_surf_, at the working electrode. Substrate potential, *E*_surf_ = −*E*_app_. (C) Stepwise events (i–iv) at each hop of the scan, with the corresponding plot of (D) *z*-displacement of the pipet meniscus from the surface (meniscus contact defined as *z* = 0 μm) and (E) synchronous potential waveform. In (C–E), (i) the tip approaches the surface with *E*_surf_ = −0.5 V upon meniscus contact; (ii) *E*_surf_ is stepped to −1.05 V for 150 ms to reduce the native surface layer on the Cu substrate; (iii) *E*_surf_ then stepped back to −0.5 V and linearly swept to −1.05 V (1 V/s); and (iv) *E*_surf_ stepped to −0.5 V, and the tip retracted away from the surface. (F) Comparison of linear sweep voltammograms from the protocol described in C–E (blue trace) to analogous LSV without the electrochemical pre-treatment step (black trace).

The sample was positioned at the center of a plastic environmental cell^49^ that allowed atmospheric control (CO_2_ or argon) (Figure 1A). The cell was made from a 6.5 cm-wide polypropylene air-tight container (HPL 931, Lock & Lock, South Korea) with 100 cm^3^ volume. A gas inlet port was made on the side of this cell by fitting a two-way Omnifit connector (Kinesis, UK) into a drilled hole with epoxy seal (Sigma-Aldrich, UK). CO_2_ or Ar was humidified to ∼60% relative humidity by continuously flowing (80 mL min^–1^) through deionized water in a threaded midget bubbler (Sigma-Aldrich, UK). The humified gas was delivered to the inlet channel via tetrafluoroethylene tubing. As the gas flux was maintained, the cell of 100 cm^3^ volume should be theoretically filled in <2 min if airtight. However, the cell was purged for an ample time of 1 h prior to the experiment to ensure efficient saturation of the cell, and the flow was maintained throughout the experiment. With the cell saturated, saturation of the nanodroplet is facilitated by the short diffusion path (the radius of the nano-droplet is ≈300 nm, *vide infra*) for the gas species. A *ca.* 10 mm-wide hole in the lid of the environmental jacket allowed translation of the tip to the appropriate position on the polycrystalline sample and gas outflow. Part of the cell lid was cut out and replaced with a 30 mm-diameter circular cover glass (Thermo Fisher Scientific, UK) and sealed in place, to enable visualization of the tip position in the area of interest on the Cu surface with a high-resolution optical camera (Pixelink, US; CompactTL 8× telecentric lenses, Edmund Optics, UK). Further details can be found elsewhere.^49^ The entire environmental cell was mounted on the *x*–*y* piezoelectric positioner (P-733.2 XY, PI, Germany).

All instrumentation used for tip positioning and current amplification was enclosed within an aluminum Faraday cage, equipped with vacuum-sealed panels (Kevothermal) and aluminum heat sinks, to minimize electrical and mechanical noise and maintain thermal equilibrium during SECCM scanning. The entire setup was placed on an optical breadboard with an active vibration isolation frame (PBI52515, PFA51507, Thorlabs, UK).

SECCM was deployed in the scan hopping mode,^28,29^ as shown in Figure 1B, to acquire spatially resolved linear sweep voltammograms on polycrystalline Cu. The hopping mode allowed for a point-by-point interrogation of the Cu substrate at an array of pre-defined locations within a grid. The potential, *E*_app_, was applied at the QRCE in the SECCM probe with respect to ground, and the current (*i*_surf_) flowing at the substrate at ground was measured using a home-built electrometer. The substrate was thus at a potential *E*_surf_ = −*E*_app_*versus* the QRCE in the tip.

The voltammetric protocol is illustrated in Figure 1C–E: (i) The tip was translated to the surface with *E*_surf_ held at −0.5 V *versus* Ag/AgCl (3.4 M KCl), which was carefully chosen to avoid the initiation of eCO_2_RR or other reduction/oxidation,^50,51^ including HER.^52^ A current change of 1.2 pA was used as a feedback parameter for stopping the nanopipette approach (*i.e.*, meniscus contact). (ii) *E*_surf_ was then immediately stepped to −1.05 V *versus* Ag/AgCl and held at that potential for 150 ms. As discussed later, the reduction pulse served to reduce the native surface oxides and hydroxides on the Cu surface.^53^ (iii) An LSV measurement was immediately recorded by sweeping *E*_surf_ from −0.5 to −1.05 V *versus* Ag/AgCl (sweep rate υ = 1 V s^–1^). Lastly, (iv) the tip was withdrawn from the surface and moved to the next scan position (Figure 1B). The tip position was recorded synchronously during the whole scan and used to generate a complementary topographical map, from the *z* position at each of the meniscus contact coordinates. Figure 1F shows that LSV collected with the pulse protocol incorporated does not show features associated with the native oxide layer on Cu.

The substrate current, *i*_surf_, was measured every 4 μs, averaged 257 times, to give a data acquisition rate of 1028 μs per point. Data acquisition and instrument control were carried out with an FPGA card (PCIe-7852R) controlled by a LabVIEW 2016 interface (National Instruments, USA) running on Warwick electrochemical scanning probe microscopy (WEC-SPM) software (www.warwick.ac.uk/electrochemistry/wec-spm). The droplet footprint was imaged with field emission SEM (FE-SEM) after scanning, and the dimensions of the wetted areas (geometric areas of the “effective” working electrode) were used to normalize the current measurements such that voltammograms, maps, and movies could be presented as current density, mA cm^–2^.

### Electron Backscatter Diffraction

EBSD measurements were performed on the samples after SECCM analysis. A Zeiss SIGMA FE-SEM instrument (Zeiss, Germany) with a Nordlys EBSD detector (Oxford Instruments, U.K.) was used. EBSD images were collected at 20 keV accelerating voltage, with the sample tilted at 70° to the detector. Note that the penetration depth is typically ∼70 nm on Cu for the acceleration voltage employed in this work.^54^ Therefore, activity correlations in this work are with the “bulk” orientation of the grains, which we consider to be reasonable for the ex situ evaluation of the post-catalyst structure, noting that Cu electrocatalysts are expected to be dynamic during eCO2RR.^22,23^ The *z*—normal direction IPF (IPF*z*) color maps were extracted from AZtech software linked with the instrument controls (AZtech, Oxford Instruments, UK). Following EBSD characterization, the AZtechICE software package (Oxford Instruments, UK) and Tango program in CHANNEL 5 software (Oxford Instruments HKL, Denmark) were used to interrogate the acquired EBSD data to extract the grain boundary lines and average grain parameters (*e.g.*, the Euler angles for deriving the average Miller indexes) of grains that were relevant for correlation with SECCM data.

## Results and Discussion

### Electroreduction Activity *Versus* Crystallographic Orientation among Low-Index Facets

We consider the onset potential region (from −0.5 to −1.05 V *vs* Ag/AgCl) where on low-index Cu facets, HER is dominant, while the products HCOO^–^ and CO account for less than 2% of the current density.^43,53,55^ Recent work evidences that only HER was observed on Cu(100) single crystals until steps and defects were incorporated.^19^ Thus, initial consideration of only low-index facets allows us to test and confirm the SECCM screening method.

**Figure 2:**
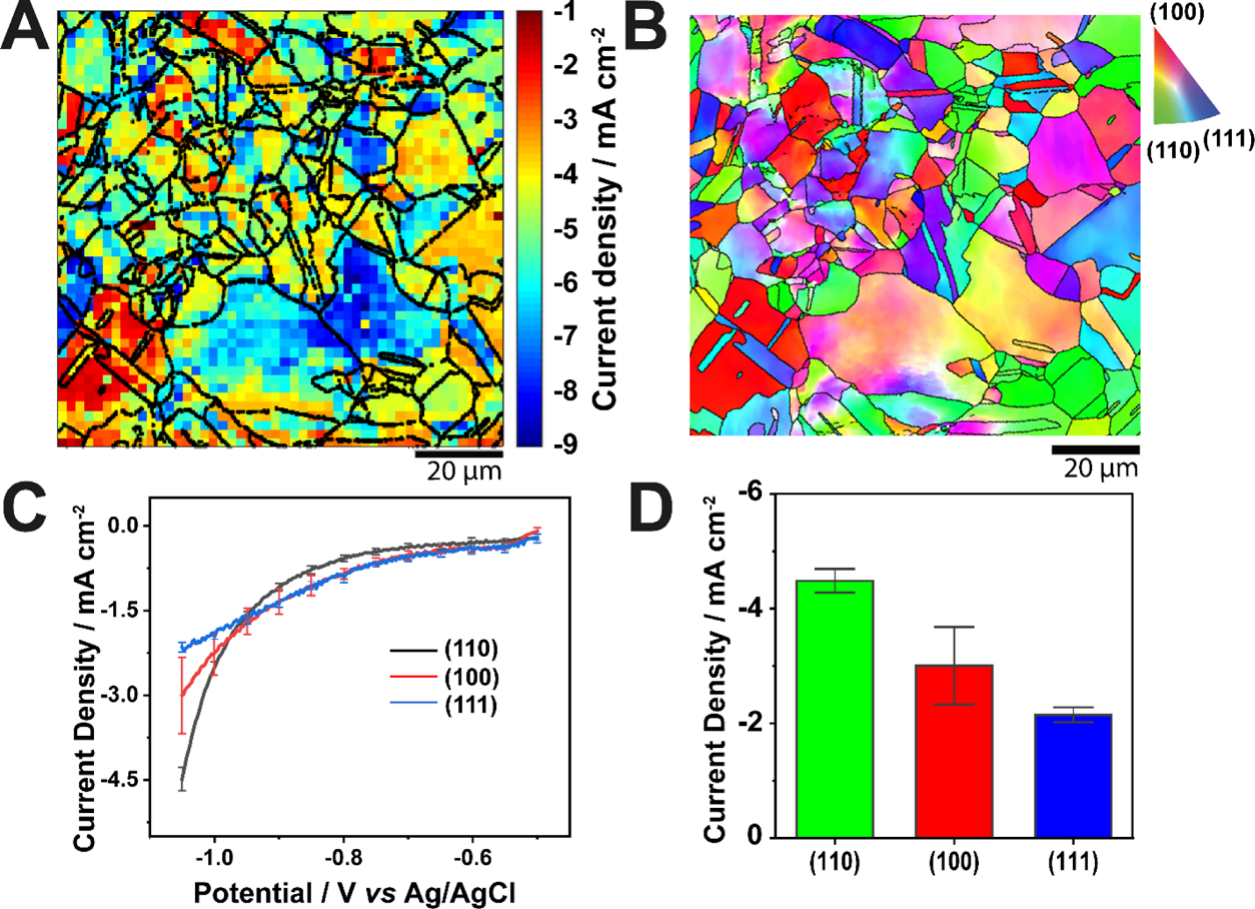
(A) Electrochemical image of a polycrystalline Cu surface, extracted from the potentiodynamic SECCM movie (Supporting Information, Movie S1) at *E*_surf_ = −1.05 V *vs* Ag/AgCl with overlay of grain boundaries (black solid lines) from (B) co-located EBSD map. The scan covers a 100 by 100 μm^2^ area composed of 2500 pixels. SECCM mapping was conducted with a nanopipette filled with 10 mM KHCO_3_ in a CO_2_-purged environmental cell. (C) Average linear sweep voltammograms obtained on grains closest to the low-index crystallographic orientations presented in blue, red, and black for (111), (100), and (110) respectively. ±1 standard deviation bars for the linear sweep voltammograms are provided at 50 mV intervals. (D) Bar chart and the current density at −1.05 V *vs* Ag/AgCl for the low-index grains in the electrochemical map (A), with *N* = 7, 17, and 24 for (111), (100), and (110) respectively.

Figure 2 presents the results of an SECCM scan (100 μm × 100 μm area) on polycrystalline Cu, acquired under a CO_2_-saturated atmosphere with a 200 nm-diameter nanopipette probe filled with the 10 mM KHCO_3_ electrolyte. The pulse-LSV scanning protocol discussed in the Materials and Methods section was used. Each pixel therefore contains an LSV measurement made by sweeping the surface potential from −0.5 to −1.05 V *versus* Ag/AgCl at a voltammetric scan rate of 2 V s^–1^, compiled as a sequence of equipotential surface current density maps for the entire potential range from −0.5 to −1.05 V (268 frames) provided in Supporting Information, Movie S1.

From the current density map at *E*_surf_ = −1.05 V (Figure 2A), clear and consistent grain-to-grain contrast can be observed. The activity reflects closely the crystallographic orientation shown by the IPF*z* color map from the EBSD analysis in Figure 2B. The SEM image (Supporting Information, Figure S1B), collected alongside the EBSD map, shows that the droplet footprints are uniform (with mean ± standard deviation of the diameter = 605 ± 61 nm). Topography maps obtained synchronously with the SECCM measurements (Supporting Information, Figure S1C,D) indicate an overall variation of topography of the order of tens of nanometers, which is on the scale expected from the dimension of the suspension employed in the last stage of the polishing procedure (0.06 μm), as described in the Materials and Methods section (*vide supra*). The substantial difference in electroreduction activity among the low-index grains, herein defined as the grains within 10° of the projection coordinates^33^ (*vide infra*) of (100), (110), and (111) orientations, is clear from the average linear sweep voltammograms in Figure 2C and the bar chart in Figure 2D. The selection of these grains is discussed in the next section. Also, the precise Euler angles, *hkl* indexes, and measures of deviation from the ideal low-index poles are supplied in Supporting Information, Table S1A, line 1–3.

The eCO_2_RR activity for the low-index planes is in the order of (110) > (100) > (111) (Figure 2D), with values of current density at *E*_surf_ = −1.05 V of 4.49 ± 0.21, 3.00 ± 0.68, and 2.15 ± 0.13 mA cm^–2^, respectively. As the overwhelmingly dominant reaction is HER, the trend of activity in neutral/alkaline media can be considered to be a subtle balance between the hydrogen binding energy and the energetics of water dissociation.^52,56−58^ The order of the reported adsorption energies of H on preferred sites on low-index Cu facets using a six-slab model^59^ agrees with the order of electroreduction activity of Cu(110) > (100) > (111) in accordance with the position of Cu on the ascending side of the volcano plot (Figure 2C, D). On the basis of water dissociation, the decreasing order of water dissociation activity on clean low-index Cu facets is (110) > (100) > (111),^60^ with Cu(110) having the lowest activation energy barrier among the low-index facets.^52,60−62^

### Electroreduction Activity *Versus* Grain Orientation among High-Index Facets

We now extend our data analysis to secondary orientations within the SECCM scan area in Figure 2, where there are approximately 60 different grains identified at the spatial scale of EBSD. Compared to low-index facets, there is an increased proportion of eCO_2_RR intermediates on high-index facets in the onset potential region, which is robustly justified by computational^44^ and experimental^63^ reports and is consistent throughout the literature.^2,7,44,53,64−66^

**Figure 3:**
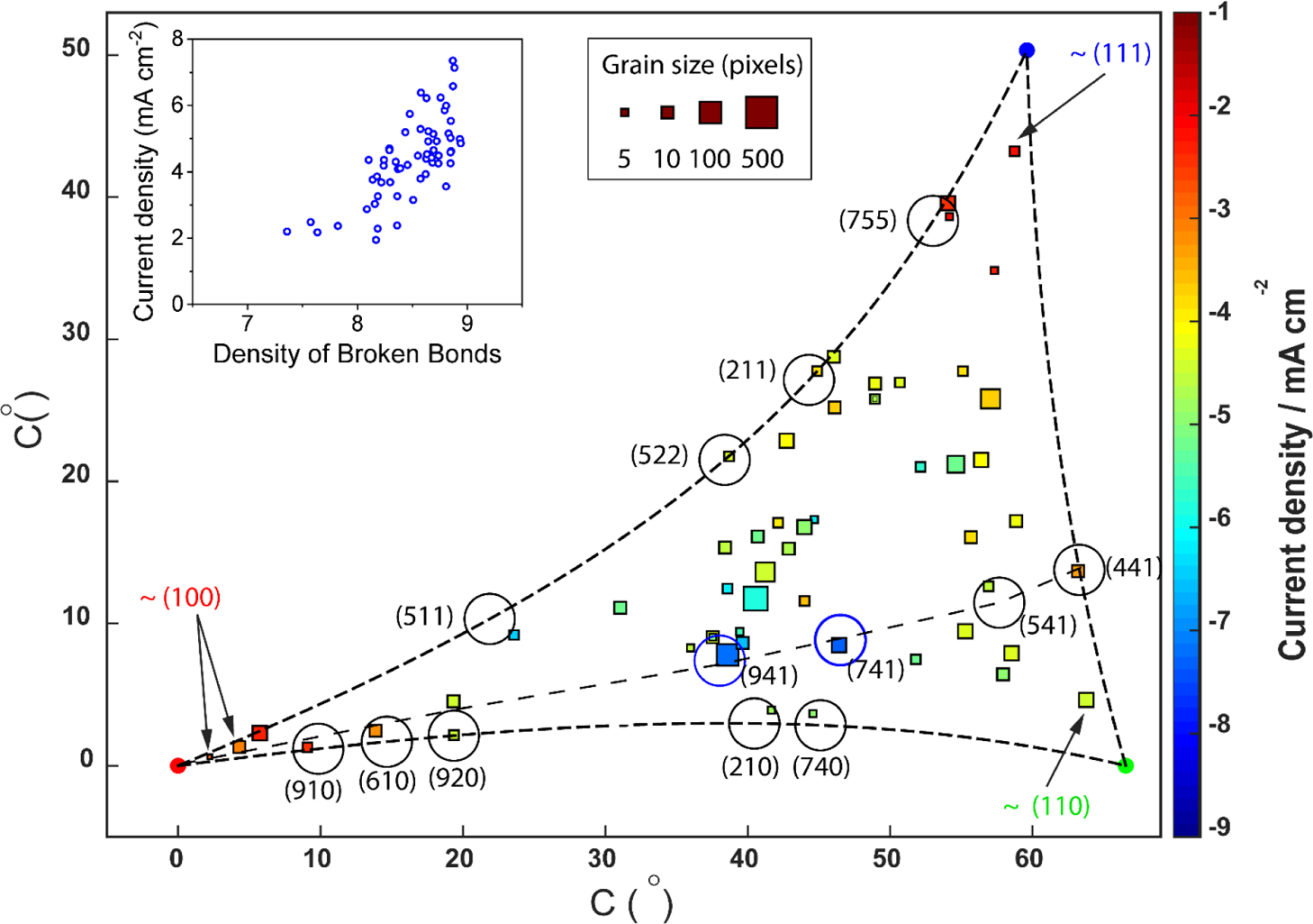
Two-dimensional projection of Cu grain orientations in an fcc crystal system correlated with electrochemical data from SECCM. Grain-resolved electroreduction current density measurements at *E*_surf_ = −1.05 V *vs* Ag/AgCl (extracted from the SECCM scan in Figure 2 and Movie S1) plotted *vs* the corresponding average grain orientation relative to the low-index orientation poles. The size of each data point is proportional to the number of SECCM pixels of that orientation according to the displayed scale. Further details of each grain within the plot can be found in Supporting Information, Table S1 (Section S4). Arrows point to the data points closest to the three low-index facets that were chosen to represent (100), (111), and (110) grains in Figure 2. Dashed lines at the boundaries and the bisecting path across the triangle cover various high-index crystallographic structures. Along these lines, the projected coordinates for specific high-index orientations are identified with black circles of a diameter of 3.0° in the projection coordinates. Hence, a data point in or on a drawn circle is considered to represent grains identical to (or within ±1.5 degrees of) the indicated orientation, with the Miller index written next to the circle. Cu(941) and Cu(741) facets are emphasized in blue. The inset shows the relationship between measured current density on the 60 grains and density of broken bonds. Detail of the calculation is provided in Supporting Information, Section S5.

To visualize differences in orientation between such grains, a two-dimensional projection was employed, representing the grains with a conformation analogous to the IPF*z*. Full details of the method development and calculation are published elsewhere.^32^ Data from Figure 2A are plotted in Figure 3, according to the surface crystallography, with the low-index grains in the face-centered cubic (fcc) crystal system, (100), (110), and (111), discussed in the previous section, shown, respectively, in red, green, and blue in the corners of the plot. The average Euler angles, *hkl* index, coordinates on the two-dimensional projection, electroreduction current density, and number of linear sweep voltammograms collected on each grain with SECCM are detailed in Supporting Information, Section S4, Table S1. Figure 3 is essentially a scatter plot in which each point represents average electroreduction activity at individual grains. The *x*–*y* coordinate of each point corresponds to the crystal orientation, the size is determined by the number of LSV measurements (pixels) collected on the grain, and the color of the point scales with electrochemical activity, that is, the measured current density at *E* = −1.05 V *versus* Ag/AgCl.

A significant observation in Figure 3 is that grains within 5° deviation from the low-index poles—(100) and (111), especially—show comparable electroreduction activity to the nearby low-index orientation. Outside of these regions, much larger electroreduction current densities are generally recorded on the high-index orientations. In a control scan, collected under Ar, where the reaction is restricted to the HER (Supporting Information, Figure S2, Movie S2, and Table S1B), less current was observed on the secondary facets within the triangular projection compared to surfaces closer to the (100) and (111) poles (see further details in Supporting Information, section S6). The latter two orientations had the lowest activity of all the surfaces assessed under CO_2_. Note further that the points on the bisection line in Figure S2 have the lowest activity, whereas under eCO_2_RR conditions, this is the region of the highest activity. Also, the overall lower current density observed in the CO_2_ scan (<10 mA cm^–2^, Figure 3) compared to Ar (up to 20 mA cm^–2^, Figure S2) is due to the inhibition of HER in CO_2_ atmospheres.^63,67^ Thus, it is reasonable to conclude that the trend observed under CO_2_ for the high-index facets shown in Figure 3 is mainly attributable to the generation of eCO_2_RR products in line with the original work of Hori^68^ and more recent work, which has shown that such high-index structures can readily transform in *operando* to produce highly active stepped surfaces.^19^ As emphasized below, a key feature of the SECCM screening method is the possibility of assessing unusual surface indexes present on polycrystalline Cu which have not been assessed before.

**Figure 4:**
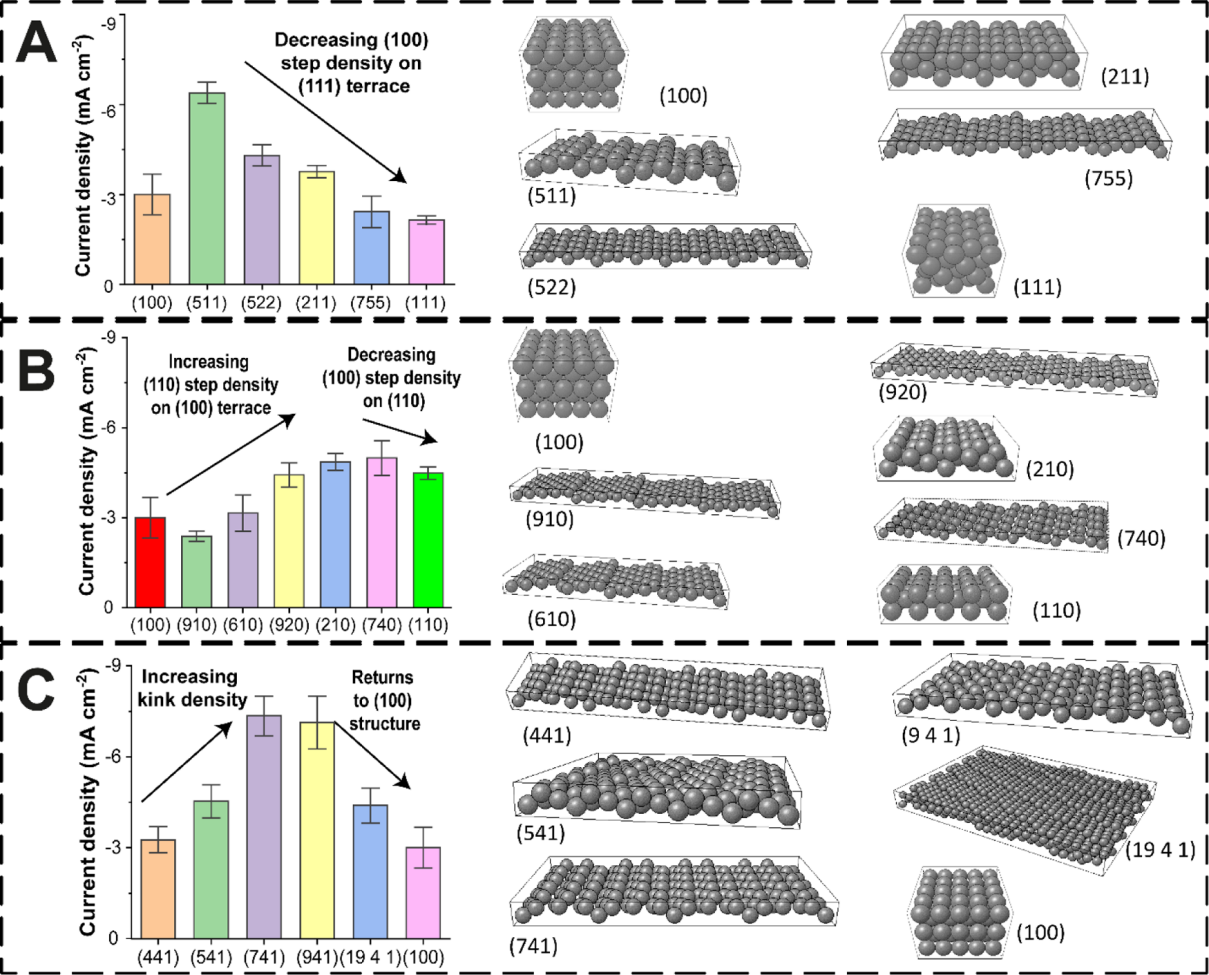
Trends of electroreduction activity on high-index Cu orientations along the two-dimensional projection of grain orientation in Figure 3. The current densities at the end of LSV (*V* = −1.05 V *vs* Ag/AgCl) are shown for (A) (100)–(111) axis, (B) (100)–(110) axis, and (C) bisecting path between (100) and (441). Rigid ball models of the crystallographic orientations identified in the bar charts are presented alongside the respective graphs from (A–C).

The inset of Figure 3 also established the correlation of the measured electroreduction current density with the density of broken bonds estimated for the grains in the triangular projection. See Section S5 of the Supporting Information for the details of the calculation. To delve more deeply into the surface structure–electrochemical activity, we identified significant points on the plot (each marked with a circle) and associated them with corresponding model crystal structures (shown in brackets beside the selected point), as displayed in Figure 4. Figure 4A shows the measured electroreduction current density of the grains between (100) and (111), which are characterized by Miller indices (*m*11), where 1 < *m*. Upon transiting from (111) toward (100), first, an increasing number of (100) steps are added to the (111) terraces.^1,4,69^ This holds up to (311), after which the structure inverts, and (111) steps on (100) terraces are observed.^69^ Electroreduction activity can be seen to rise with increasing (111)-step density, that is, from (100) to (511) and also with increasing (100)-step density: from (111) to (522). This suggests that the most stepped high-index grains are most active for electroreduction. Specifically, Cu(511), which is identified as the orientation with the highest reduction current density on this path (Figure 4A), has one (111) step every three atoms along the (100) terrace {*i.e.*, analogous to Cu[S]—[3 (100) × (111)]}. Interestingly, Cu(511) is about 50% more active than its counterpart, Cu(211), which has similar step density but with (100) steps on (111) terraces {*i.e.*, Cu[S]—[3 (111) × (100)]}. This suggests that (100) steps are more active compared to (111) steps. This trend is consistent with the variation in electrode potential and C_2_ product yield in current-controlled experiments at −5 mA cm^–2^ current density in a series of single crystal electrodes.^68^

In Figure 4B, the trend of activity from (100) to (110), with Miller indices (*0n1*), where 0 < 1 < *n*, is presented. Upon moving from (100) toward (110), steps of (110) are gradually added to the (100) terraces. This (110) step is unique as it is composed of kinks, unlike steps of (111) and (100), up to a maximum kink density of the (210) surface.^4,68,69^ Electroreduction activity can be seen to increase in the same manner as described above. In contrast, structures between (210) and (110) have (100) steps and (110) terraces. Cu(740) is in this category and is where we observed the highest electroreduction current density within the grain selection considered. For all three boundary axes, the two contributing low-index planes have the lowest activity, except for orientations on the (111)–(110) axis for which (110) is most active. We deduce that enhanced electroreduction activity is observed at high-index facets and scales with the step density and surface roughness.

The grains with the overall highest electroreduction current density are Cu(941) and Cu(741) (identified with blue circles in Figure 3), which are about 7–8° shifted from the middle of the (100)–(110) axis toward the center of the triangle. A dashed line that originates from (100) and bisects the (111)–(110) axis at point (441) is drawn on the grain orientation projection in Figure 3. With reference to Figure 4C, starting from the stepped (441) index, the kink density increases gradually along this line such that the points in the middle of the stereographic triangle, in the vicinity of (741) and (941) orientations, present complex atomic arrangements.^69^ The 3D rigid ball models of the two orientations show the most closely packed arrangement of the kink atoms. Further along the zone axis, the surface becomes smoother, approaching the (100) surface. Thus, introducing kinks to stepped surfaces is critical to their catalytic performance, in agreement with a previous report on eCO_2_RR for a series of high-index single crystal platinum electrodes.^70^ Interestingly, Cu surfaces similar to (741) and (941) have received little attention in experimental and density functional theory studies, although the data presented herein suggest that they are worthy of further analysis as high-activity surfaces.

### Fingerprinting the Native Surface Layer on Polycrystalline Cu with Voltammetric SECCM

**Figure 5:**
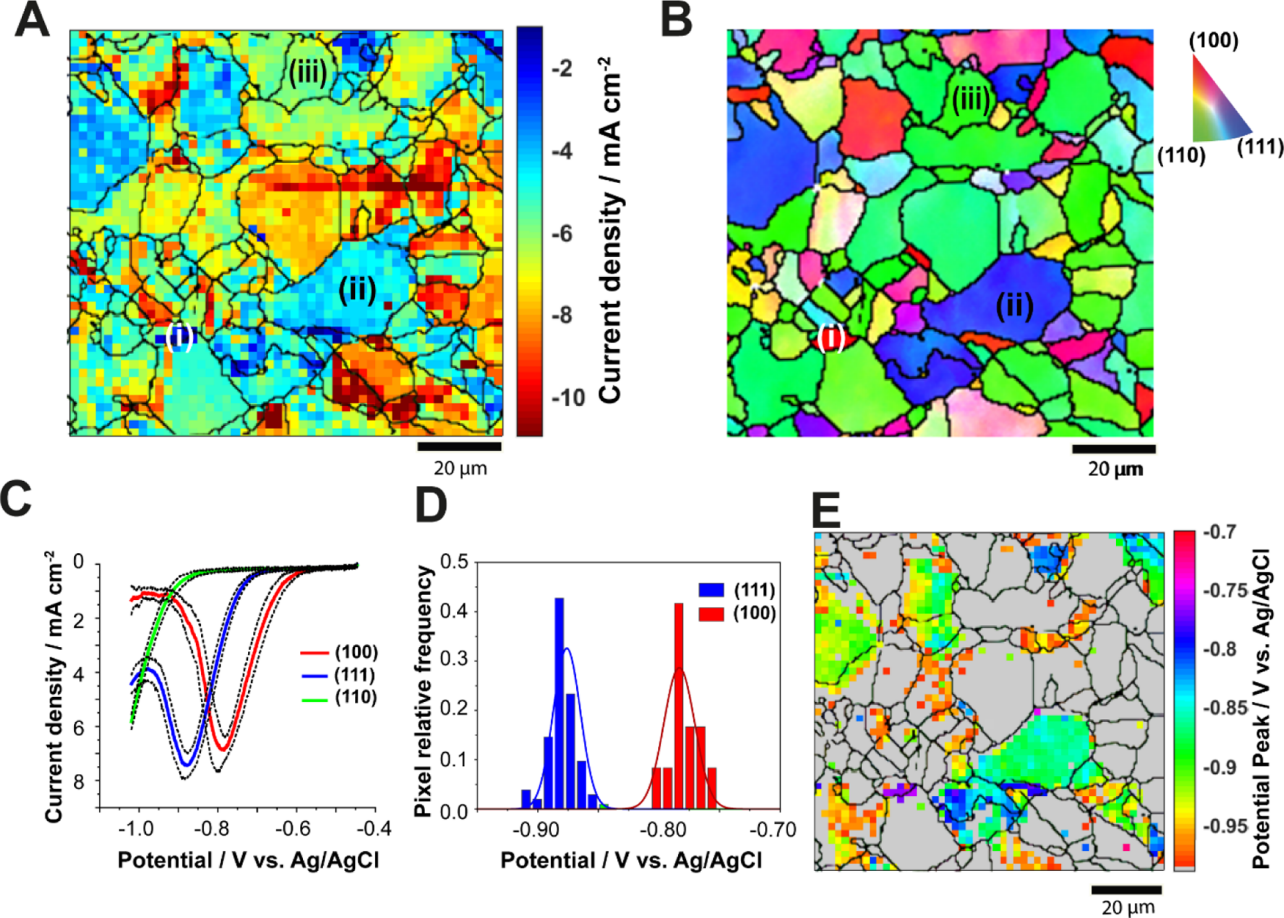
(A) Electrochemical image of the measured current density on a polycrystalline Cu surface, extracted from the potentiodynamic SECCM movie (Movie S3) at *E* = −1.05 V *vs* Ag/AgCl. (B) Co-located EBSD map. The scan covers a 100 × 100 μm^2^ area comprising 2500 pixels. SECCM mapping was conducted with a nanopipette filled with 10 mM KHCO_3_ in a CO_2_ environmental cell. (C) Averaged linear sweep voltammograms collected on low-index grains [(100), (111), and (110)] which are identified as (i, ii, and iii) in (A) and (B). Shown on linear sweep voltammograms are the average current density–voltage curves (solid lines) ± one standard deviation (dotted lines), obtained from 13, 78, and 39 independent linear sweep voltammograms selected from grains (100), (111), and (110), respectively. (D) Histograms of peak position values for the Cu(111) and (100) LSV data from (C). (E) Spatially resolved peak potential map, extracted from individual linear sweep voltammograms. In this map, pixels with peakless linear sweep voltammograms are colored gray.

In this section, we clarify the importance of the pulse phase in the SECCM protocol detailed in the Materials and Methods section for stripping the native passive layer on the Cu surface. This work also illustrates how SECCM can be used to assay the electrochemical properties of surface layers on electrodes. Presented in Figure 5 are the results of a 100 × 100 μm^2^ SECCM LSV scan in a CO_2_ environmental cell for which the potential pulse phase was omitted. Figure 5A shows *i*_surf_ at *E*_surf_ = −1.05 V *versus* Ag/AgCl, taken from a potentiodynamic movie for the entire potential range of *E*_surf_ = −0.45 to −1.05 V *versus* Ag/AgCl (Supporting Information, Movie S3). A corresponding co-located EBSD map is presented in Figure 5B. By comparing Figure 5A with Figure 5B, surface grain dependency of the electrochemical response is evidenced.

Figure 5C shows the average linear sweep voltammograms (*N* = 13, 78, and 39) selected from the grains closest to the primary low-index orientations, (100), (111), and (110), marked as i, ii, and iii, respectively, in Figure 5A,B. It is clear from Figure 5C that the entire voltammetric profiles are unique for each low-index grain. Distinct reduction peaks attributable to the stripping of the native hydroxide layer of Cu (vide infra) are observed on the linear sweep voltammograms for Cu(100) and Cu(111) at −0.78 and −0.88 V *versus* Ag/AgCl, respectively (Figure 5C,D). However, a reduction peak is not observed on grain (110); the reduction current density magnitude continues to increase within the potential range studied. This is because the potential region for native hydroxide stripping on Cu(110) overlaps with the voltammetric onset of CO_2_ reduction/hydrogen evolution.^12,71^ The grain dependency of the peak position is consistent for the whole scan and can be supported by maps of the peak potential (Figure 5E) and crystallographic orientations obtained from EBSD (Figure 5B).

**Figure 6:**
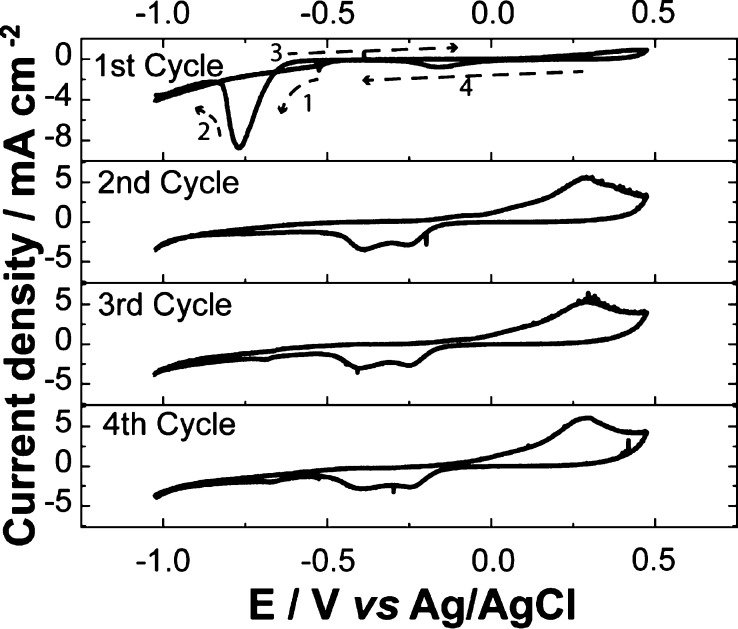
Cyclic voltammograms on polycrystalline Cu from a single-point SECCM measurement. The experiment was performed with a nanopipette filled with 10 mM KHCO_3_ in an Ar environmental cell. The potential was swept from 0.5 to −1.05 V *vs* Ag/AgCl at a scan rate of 1 V s^−1^ for four consecutive cycles. The direction of potential sweeps is noted with dashed arrows on the first cycle.

To further understand the reduction, we studied the native layer stripping in an argon atmosphere with four sequential cyclic voltammetry (CV) cycles at a single point on the Cu surface (Figure 6).^33^ In the first cycle, a characteristic reduction peak at −0.78 V was observed, similar to the average curve on Cu(100) (Figure 5C). On the second and subsequent cycles, the reduction peak disappeared, and (quasi-)reversible Cu surface redox processes occurred between −0.25 and −0.40 V.^12^ This rules out the possible correlation of the reduction peak at −0.78 V with reduction processes of electrochemically formed CuO and Cu_2_O. Moreover, the reduction peak at −0.78 V is related to the initial state of the surface rather than a reversible adsorption/desorption of electrolyte species (e.g., HCO_3_^–^, SO_4_^2–^, etc.).^11,72,73^ It is also noteworthy that the peak is observed in a CO_2_ (Figure 5) and argon (Figure 6) environment.

The reduction process from −0.78 to −0.88 V has been identified as the reduction of soluble precursors, namely, Cu(OH)_2_, on Cu surfaces.^53,74−76^ Cu(OH)_2_, together with Cu_2_O and CuO, is the product of native oxidation of Cu in an ambient atmosphere.^51^ Up to 2.5 nm-thick Cu_2_O and Cu(OH)_2_ can be formed on Cu surfaces within 1 h when exposed to ambient air, while CuO formation is much slower.^51^ The charge density of the peak in the first CV cycle in Figure 6 is about 657 μC cm^–2^ which corresponds to ∼few monolayers of the native oxide. The presence of Cu(OH)_2_ on as-prepared Cu and its removal by electrochemical reduction potential were further corroborated with X-ray photoelectron spectroscopy (XPS) measurement (See Supporting Information, Section S7).

As seen from the results of Figure 6 and the surface composition characterization, the maps shown in Figure 5 reveal the crystallographic orientation dependence of the reduction potential of the native Cu(OH)_2_ adlayer on low-index Cu surfaces: (100) > (111) > (110). The surface oxides are removed at potentials more positive than the onset of HER and eCO_2_RR on Cu, especially on Cu(100) and Cu(111). There is debate as to whether the product selectivity for eCO_2_RR reduction (at more negative potentials than in this work) is from participation of remnant oxide species^16,77^ or from structural/morphological transformations that accompany the reduction of the oxide layers.^17,53,78^ Our electrochemical results lend credence to the latter hypothesis, as the oxide signal is not observed after the first cycle. If the oxide does remain, it is at an electrochemically undetectable level.

## Conclusions

We have implemented a pseudo-single crystal screening approach, engaging co-located voltammetric SECCM and EBSD, to reveal surface structure–activity correlations for eCO_2_RR at multiple sites across a polycrystalline Cu surface. Focusing on the onset potential region, we observed the activity trend to be (111) < (100) < (110) among the low-index facets, under CO_2_ electroreduction conditions. However, product analysis from past studies suggests that H_2_ evolution is the dominant process on the primary orientations of Cu. For high-index facets, eCO_2_RR is more significant, and analyses of *ca.* 60 different grains across the secondary orientations, collectively visualized on a stereographic triangle, unambiguously reveal that the density and nature of steps and kinks introduced to the Cu catalyst surface are critical to achieving enhanced eCO_2_RR activity. The electroreduction current scales with the density of broken bonds of the surfaces. The overall activity trend is as follows: (111) < (100) < Cu(S)—[*n* (111) × (111)] < (110) < Cu(S)—[*n* (111) × (100)] < Cu(S)—[*n* (100) × (111)] < Cu(S)—[*n* (100) × (110)] < Cu(S)—[*n* (441) × (100)]. The most active orientations among the grains studied in this work are Cu(941) and Cu(741), which are highly kinked step surfaces located in the middle of the stereographic triangle. These Cu(941) and Cu(741) surfaces, derived from stepped Cu(441)—a member of the Cu(S)—[*n* (111) × (111)] category and rarely studied for electroreduction performance, may be crucial to achieving improved electroreduction activity on Cu and are worth further study. Lastly, the electrochemical stripping of the native Cu(OH)_2_ passive layer was shown to be facet-controlled, and the order of relative ease of stripping from easiest is (100) > (111) > (110). This fingerprinting phenomenon further presents evidence of the important role played by crystallographic facets in the electrochemical behavior of Cu electrocatalysts.

Altogether, this study enables the screening and analysis of a large number of surface structures, identifying candidate surfaces for further study and providing information that could be used to aid the rational design of improved catalysts for CO_2_ reduction. This study motivates a broader exploration of structural engineering as a method to improve electrocatalytic performance. A further direction will be the implementation of on-line product analysis and instrumental coupling of SECCM with in situ surface spectroscopy techniques to enable selectivity measurements in the study of complex reaction systems like eCO_2_RR.

## Supporting Information Available

Additional methods; electrochemical movie captions; SEM images and SECCM topography maps of areas scanned with SECCM; *hkl* detail of grains in SECCM scan area: estimation of density of broken bonds; results of the SECCM scan under argon; XPS results; and SI references (PDF)
Electrochemical movie for the eCO_2_RR scan in a CO_2_-purged environmental cell (AVI)
Electrochemical movie for the HER scan in a Ar-purged environmental cell (AVI)
Electrochemical movie for native adlayer stripping (AVI)

## Supplementary Material

cs2c01650_si_001.pdf

cs2c01650_si_002.avi

cs2c01650_si_003.avi

cs2c01650_si_004.avi
