# Supplementary material for: Screening Surface Structure–Electrochemical Activity Relationships of Copper Electrodes under CO2 Electroreduction Conditions
Source: ACS Catal. 2022 May 19;12(11):6578–88. doi: 10.1021/acscatal.2c01650 (PMC9171721; doi:10.1021/acscatal.2c01650)
Supplement: Supplementary file 1 — cs2c01650_si_001.pdf [file cs2c01650_si_001.pdf]

Supporting Information

Screening surface structure-electrochemical activity relationships of copper electrodes under CO_2 electroreduction conditions

Oluwasegun J. Wahab,[1] Minkyung Kang,[2,] Enrico Daviddi,[1] Marc Walker,[3] and Patrick R. Unwin.[1,*]*

[1] Department of Chemistry, University of Warwick, Coventry CV4 7AL, U.K.; [2] Institute for Frontier Materials Deakin University, Geelong, VIC 3217, Australia; [3] Department of Physics, University of Warwick, Coventry CV4 7AL, U.K

* Corresponding author. Email address: p.r.unwin@warwick.ac.uk (PRU) and m.kang@deakin.edu.au (MK)

Contents

S1. Methods …………………………………………...….….…………………. S3

S2. Electrochemical movie captions ……………………..….……….………........ S5

S3. SEM images and SECCM topography maps of areas scanned with SECCM..... S6

S4. *hkl* detail of grains in SECCM scan area ………………………………......…... S7

S5. Estimation of density of broken bonds …………………………………....…. S10

S6. Results of SECCM scan under Argon ………………………………………... S11

S7. XPS results ……………………………………………………………………. S13

S8. References ……………………………………………………………………. S16

S1. Methods

Scanning electron microscopy: SEM images were obtained with a Zeiss SUPRA 55-VP FEGSEM and Zeiss SIGMA FE-SEM with In Lens detector. SEM images were collected at either 5 keV or 20 keV when collected alongside electron backscatter microscopy images.

X-ray photoelectron spectroscopy (XPS): XPS data were collected at the Warwick Photoemission Facility, University of Warwick, United Kingdom, on a Kratos Axis Ultra DLD spectrometer which had a base pressure below 1 x 10^{-10} mbar. A polycrystalline Cu sample, prepared as described in the main text and stored under vacuum, underwent a first XPS characterization without further treatment. The sample was then immediately subjected to an electrochemical reduction pulse of -1V vs Ag/AgCl for 1 hour, in a three-electrode macroscale setup using a graphite rod counter electrode, before subsequent XPS measurement. The samples were attached to electrically conductive carbon tape and mounted onto a sample bar that was then loaded into the main chamber of the spectrometer.

XPS measurements were performed in the main analysis chamber, with the sample being irradiated with a monochromated Al Kα x-ray source (hν = 1486.7 eV). The measurements were conducted at room temperature and at a take-off angle of 90° with respect to the surface parallel direction. The core level spectra were recorded using a pass energy of 20 eV (resolution approx. 0.4 eV), from an analysis area of 300 microns x 700 microns. The work function and binding energy scale of the spectrometer were calibrated using the Fermi edge and $3d_{5/2}$ peak recorded from a polycrystalline Ag sample prior to the commencement of the experiments. To prevent surface charging, the surface was flooded with a beam of low-energy electrons throughout the experiment and this necessitated recalibration of the binding energy scale. To achieve this, the main C-C/C-H component of the C 1s spectrum was referenced to 284.8 eV. The data were analyzed with the

CasaXPS software package, using Shirley backgrounds and mixed Gaussian-Lorentzian (Voigt) lineshapes. For compositional analysis, the analyzer transmission function was determined using clean metallic foils to determine the detection efficiency across the full binding energy range.

S2. Electrochemical Movie Captions

Movie S1: Electrochemical (potentiodynamic) movie of voltammetric SECCM measurements on a polycrystalline Cu substrate under CO_2 condition for the results discussed in Figure 2 of the main text. The pulse-LSV protocol (as discussed in the Methods section of the main text) was used for the scan acquisition. The movie shows a series of electrochemical frames that correspond to current maps of the Cu substrate as the potential, E_{surf}, is swept from -0.45 V to – 1.05 V *vs* Ag/AgCl. Scan area is 100 µm by 100 µm and consists of 2,500 pixels (2 µm pitch).

Movie S2: Electrochemical (potentiodynamic) movie of voltammetric SECCM measurements on a polycrystalline Cu substrate under Ar for results presented in SI, Section S6. The pulse-LSV protocol (as discussed in the Methods section of the main text) was used for the scan acquisition. The movie shows a series of electrochemical frames that correspond to current maps of the Cu substrate as the potential, E_{surf}, is swept from -0.45 V to – 1.05 V *vs* Ag/AgCl. Scan area is 100 µm by 100 µm and consists of 2,500 pixels (2 µm pitch).

Movie S3: Electrochemical (potentiodynamic) movie of voltammetric SECCM measurements on a polycrystalline Cu substrate without pulse treatment (as discussed in Figure 5 of the main text). LSVs correspond to the electrochemical stripping of native passive $Cu(OH)_2$ layer on Cu. The movie shows a series of electrochemical frames that correspond to current maps of the Cu substrate as the potential, E_{surf}, is swept from -0.45 V to – 1.05 V *vs* Ag/AgCl. Scan area is 100 µm by 100 µm and consists of 2,500 pixels (2 µm pitch).

S3. SEM images and SECCM topography maps of areas scanned with SECCM

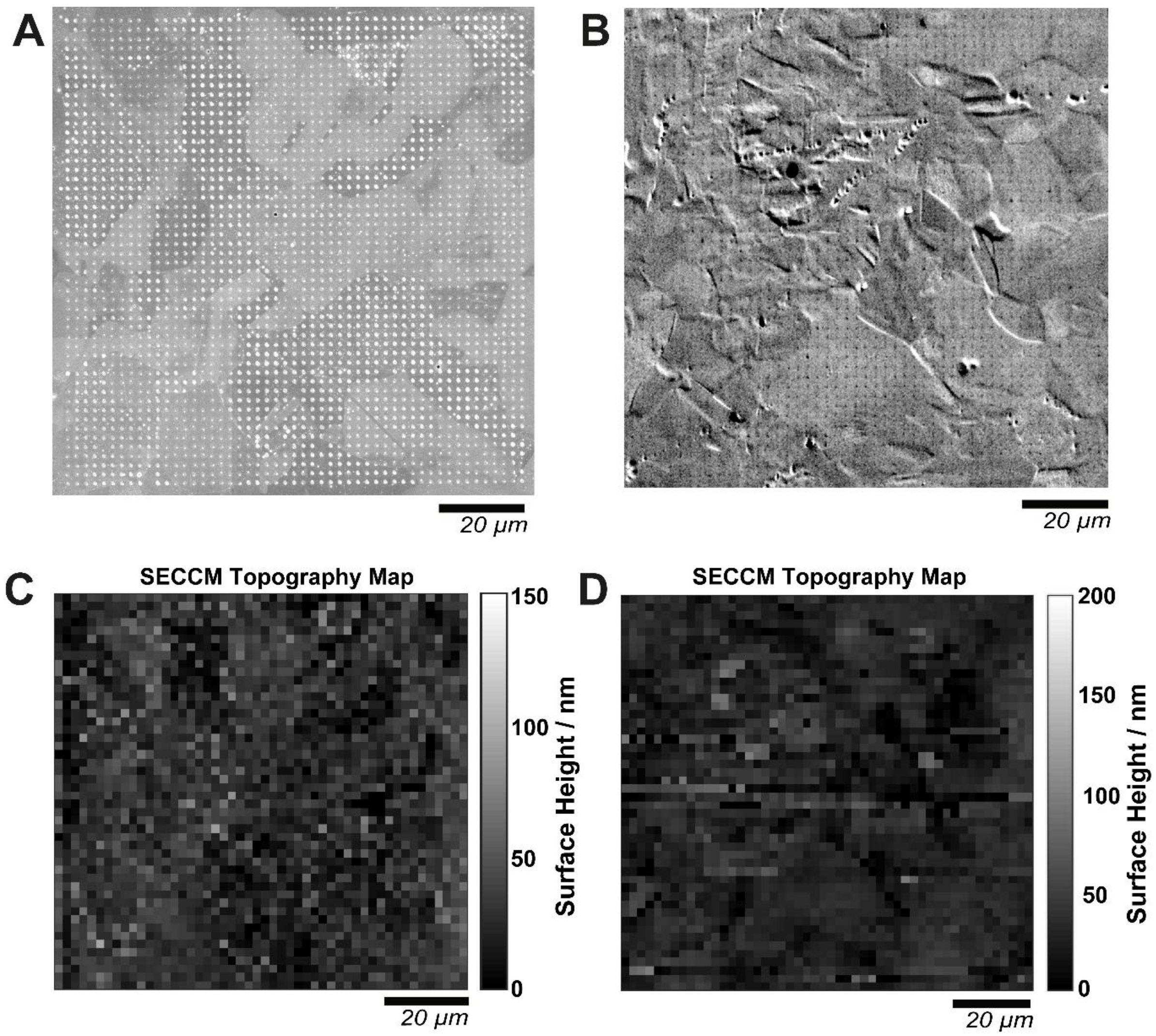

Figure S1: SEM images visualizing the footprints of the individual droplet cell for the voltammetric SECCM scan discussed in: (A) Figure 5 of the main text (Movie S1); and (B) Figure 2 of the main text (Movie S3). The droplet diameter was 835 ± 69 nm for sample size, n = 564 (A) and 605 ± 61 nm based on 25 individual measurements (B). SEM images confirm droplet wetting to be regular throughout the scan without overlap, and independent of grain orientation. The SEM image in (A) was collected at 5 keV while the one in (B) was collected alongside EBSD at 20 keV with 70° sample tilt. (See methods section of main text and SI, section S1). Topography maps synchronously acquired in SECCM scan presented in: (C) presented in Figure 5 of main text (Movie S1); and (D) Figure 2 of the main text (Movie S3). Topography maps in (C) and (D) correspond to SEM images in (A) and (B) respectively.

S4. *hkl* detail of grains in the SECCM scan area

Table S1-A: Details of crystal grains in SECCM eCO_2RR scan presented in Figure 5, main text. The grains selected as representations of low index facets are prsensented first in ID numbers 1-3, and highlighted, in color red, blue, and green for (100), (111), and (110) respectively.

ID number	Average Euler angles (°)			Average miller indices			Coordinates in the space of the grains Angle from low index planes (°)			Coordinates of Projection 2D crystallographic projection		E_{surf} at -1.05 V *vs* Ag/AgCl (mA/cm^2)	Number of pixels
	φ_1	Φ	φ_2	*h*	*k*	*l*	From 100 X	From 110 Y	From 111 Z	C_1	C_2		
1	113.9	1.4	64.3	1.000	0.022	0.011	1.4	43.7	53.4	0.00	0.00	2.87	27
2	138.0	46.4	45.4	0.689	0.516	0.509	46.4	31.6	8.3	56.25	43.77	2.49	12
3	271.0	43.4	5.3	0.727	0.684	0.063	43.4	4.0	31.7	60.44	7.84	4.49	32
4	15.7	26.7	33.1	0.892	0.376	0.246	26.8	26.1	28.9	35.07	13.81	4.52	7
5	140.4	31.3	59.7	0.855	0.448	0.262	31.3	22.9	25.4	42.94	14.25	6.22	5
6	41.6	23.4	17.9	0.918	0.378	0.122	23.4	23.6	35.1	33.29	7.83	4.62	4
7	131.7	24.5	71.1	0.910	0.392	0.135	24.5	23.0	34.0	41.91	8.05	4.26	5
8	320.7	39.4	80.1	0.773	0.625	0.109	39.4	8.7	29.5	60.44	7.84	4.28	2
9	47.2	27.9	26.8	0.884	0.418	0.211	27.9	23.0	29.2	42.94	14.25	4.49	18
10	156.1	34.1	10.2	0.828	0.552	0.100	34.1	12.6	31.3	54.27	8.40	5.15	10
11	137.3	30.7	59.9	0.860	0.441	0.256	30.7	23.1	26.0	42.94	14.25	4.92	22
12	131.7	24.5	71.1	0.910	0.392	0.135	24.5	23.0	34.0	41.91	8.05	4.59	69
13	131.7	24.5	71.1	0.910	0.392	0.135	24.5	23.0	34.0	41.91	8.05	5.02	14
14	305.4	21.0	58.6	0.934	0.306	0.187	21.0	28.8	34.6	35.07	13.81	5.14	5
15	172.3	35.3	40.1	0.817	0.441	0.372	35.3	27.2	19.8	47.52	23.94	3.69	27
16	284.5	38.6	8.0	0.781	0.618	0.086	38.6	8.3	30.9	60.44	7.84	4.93	3
17	146.3	5.8	3.6	0.995	0.101	0.006	5.8	39.2	50.5	9.85	1.19	2.38	7
18	227.2	38.7	53.2	0.780	0.501	0.375	38.7	25.1	17.0	53.62	24.20	4.36	6
19	171.8	32.4	48.9	0.845	0.403	0.352	32.4	28.1	22.5	43.72	20.83	4.08	9
20	124.1	2.8	60.6	0.999	0.042	0.024	2.8	42.6	52.1	0.00	0.00	3.03	6
21	173.4	37.6	38.8	0.792	0.476	0.382	37.6	26.3	17.7	47.52	23.94	4.19	34
22	167.2	39.6	63.0	0.771	0.568	0.289	39.6	18.8	20.0	53.62	24.20	5.19	22
23	136.4	4.2	45.3	0.997	0.052	0.052	4.2	42.1	50.5	0.00	0.00	3.26	22
24	19.1	37.2	37.5	0.797	0.479	0.368	37.2	25.5	18.4	47.52	23.94	4.70	23
25	59.8	12.4	18.3	0.977	0.203	0.067	12.4	33.4	43.9	21.38	6.40	4.39	23

**26**	19.1	37.2	37.5	0.797	0.479	0.368	37.2	25.5	18.4	47.52	23.94	4.65	23
**27**	20.2	29.2	2.3	0.873	0.487	0.019	29.2	15.9	37.2	40.46	2.97	4.99	24
**28**	323.8	51.2	48.8	0.627	0.586	0.513	51.2	31.0	4.7	56.25	43.77	2.20	24
**29**	321.2	46.7	53.3	0.686	0.584	0.434	46.7	26.1	10.3	19.36	2.11	2.37	24
**30**	300.2	12.5	0.3	0.976	0.217	0.001	12.5	32.5	46.4	41.91	8.05	4.42	25
**31**	42.1	25.8	18.7	0.900	0.412	0.140	25.8	21.9	33.0	42.94	14.25	5.53	25
**32**	141.3	29.5	61.5	0.871	0.432	0.235	29.5	22.9	27.4	42.94	14.25	4.65	25
**33**	216.8	37.6	28.8	0.792	0.535	0.294	37.6	20.2	20.6	53.62	24.20	5.75	26
**34**	175.5	35.9	45.0	0.810	0.414	0.414	35.9	30.0	18.9	47.73	31.03	3.77	26
**35**	69.9	29.6	32.5	0.869	0.417	0.265	29.6	24.6	26.4	42.94	14.25	3.93	26
**36**	265.6	16.1	53.6	0.961	0.224	0.165	16.1	33.1	38.8	21.38	6.40	6.39	27
**37**	50.2	25.9	63.4	0.899	0.391	0.196	25.9	24.2	30.9	42.94	14.25	6.24	27
**38**	255.5	43.2	29.4	0.729	0.597	0.335	43.2	20.4	16.5	53.62	24.20	3.68	28
**39**	41.0	29.8	45.0	0.868	0.351	0.351	29.8	30.4	25.0	36.67	20.09	4.30	28
**40**	23.0	25.0	75.1	0.906	0.409	0.109	25.0	21.6	34.7	41.91	8.05	7.14	28
**41**	345.9	27.0	23.4	0.891	0.417	0.180	27.0	22.4	30.8	42.94	14.25	5.86	29
**42**	96.8	42.1	70.0	0.742	0.630	0.229	42.1	14.0	22.4	59.28	15.88	4.21	29
**43**	174.2	37.0	45.4	0.799	0.428	0.422	37.0	29.8	17.8	47.73	31.03	4.36	29
**44**	259.1	37.0	12.6	0.798	0.588	0.132	37.0	11.5	28.8	54.27	8.40	4.25	30
**45**	319.8	3.9	48.3	0.998	0.051	0.046	3.9	42.1	50.8	0.00	0.00	2.29	30
**46**	28.9	42.2	33.0	0.741	0.564	0.366	42.2	22.7	15.4	53.62	24.20	3.86	31
**47**	258.7	30.4	13.4	0.863	0.492	0.117	30.4	16.7	31.8	41.91	8.05	7.35	31
**48**	8.7	25.8	16.6	0.900	0.417	0.124	25.8	21.3	33.7	41.91	8.05	6.58	31
**49**	40.8	38.9	20.7	0.778	0.588	0.222	38.9	15.1	23.6	54.01	16.17	5.29	32
**50**	342.6	29.2	69.3	0.873	0.457	0.173	29.2	19.9	29.9	42.94	14.25	5.99	32
**51**	161.2	41.2	64.1	0.753	0.592	0.288	41.2	18.0	19.5	53.62	24.20	4.11	33
**52**	342.6	29.2	69.3	0.873	0.457	0.173	29.2	19.9	29.9	42.94	14.25	3.56	33
**53**	310.6	45.7	76.0	0.698	0.694	0.174	45.7	10.0	25.3	62.48	18.31	3.26	33
**54**	40.8	38.9	20.7	0.778	0.588	0.222	38.9	15.1	23.6	54.01	16.17	3.79	34
**55**	296.6	27.2	87.1	0.890	0.456	0.023	27.2	17.9	37.8	40.46	2.97	4.86	34
**56**	15.7	39.0	16.1	0.777	0.605	0.175	39.0	12.3	26.0	54.01	16.17	4.53	34
**57**	167.8	28.4	57.8	0.880	0.402	0.253	28.4	25.0	27.6	42.94	14.25	5.22	35
**58**	79.0	46.0	43.7	0.694	0.520	0.497	46.0	30.8	8.8	56.77	36.54	2.18	35
**59**	265.4	8.8	9.9	0.988	0.151	0.026	8.8	36.3	47.7	9.85	1.19	3.15	35
**60**	334.9	3.2	34.4	0.998	0.046	0.031	3.2	42.4	51.6	0.00	0.00	1.95	36

Table S1-B: Details of crystal grains in the HER SECCM scan presented in SI, Figure S6. The grains closest to the low index facets in this scan are prsensented first in ID numbers 1-3, and highlighted, in color red, blue, and green for (100), (111), and (110) respectively.

ID number	Average Euler angles (°)			Average miller indices			Coordinates in the space of the grains Angle from low index planes (°)			Coordinates of Projection 2D crystallographic projection		E_{surf} at -1.05 V *vs* Ag/AgCl (mA/cm^2)	Number of pixels
	φ_1	Φ	φ_2	*h*	*k*	*l*	From 100 X	From 110 Y	From 111 Z	C_1	C_2		
**1**	200.3	6.6	6.36	0.993	0.114	0.013	6.5	38.5	50.0	10.14	1.44	20.98	34
**2**	95.7	46.6	55.17	0.687	0.596	0.415	46.6	24.8	11.3	57.86	33.29	10.61	5
**3**	302.7	31.3	2.6	0.854	0.520	0.023	31.3	13.7	36.2	47.76	3.72	3.31	5
**4**	181.2	26.2	81.2	0.897	0.437	0.067	26.2	19.4	36.0	40.56	5.84	3.68	19
**5**	32.9	26.9	9.2	0.892	0.447	0.072	26.9	18.9	35.5	41.51	6.11	3.80	1
**6**	116.6	22.1	69.8	0.926	0.354	0.130	22.2	25.2	35.5	34.04	8.55	2.92	8
**7**	359.7	35.5	37.3	0.814	0.461	0.353	35.5	25.6	19.9	47.31	24.16	11.41	120
**8**	167.7	37.3	52.2	0.795	0.479	0.371	37.3	25.7	18.1	49.01	26.07	7.02	14
**9**	25.5	40.3	29.6	0.762	0.562	0.320	40.3	20.5	18.3	54.51	23.69	10.36	34
**10**	213.9	15.8	37.3	0.962	0.217	0.165	15.8	33.5	39.1	23.00	9.14	13.14	5
**11**	32.9	26.9	9.2	0.892	0.447	0.072	26.9	18.8	35.5	41.51	6.11	3.41	15
**12**	167.6	37.2	52.3	0.796	0.478	0.370	37.2	25.6	18.2	48.94	25.97	6.07	8
**13**	25.6	39.7	29.3	0.769	0.557	0.313	39.7	20.3	18.8	54.07	23.02	9.42	3
**14**	178.0	34.2	63.5	0.827	0.503	0.250	34.2	19.8	24.1	49.09	17.21	3.20	30
**15**	215.2	16.5	36.27	0.959	0.229	0.168	16.5	32.8	38.4	24.11	9.41	14.26	22
**16**	124.0	37.8	74.3	0.790	0.590	0.166	37.8	12.5	26.7	55.68	11.86	3.22	70
**17**	192.9	21.3	28.38	0.931	0.320	0.173	21.3	27.7	34.6	32.01	10.53	6.30	6
**18**	57.18	43.2	20.99	0.729	0.639	0.245	43.2	14.7	21.4	59.46	18.70	9.47	85
**19**	202.9	6.48	3.63	0.999	0.112	0.007	6.6	49.7	38.5	10.34	1.64	20.18	3
**20**	57.1	43.5	20.97	0.725	0.642	0.246	43.5	21.2	14.7	59.73	18.87	9.20	37
**21**	322.2	34.3	47.38	0.826	0.415	0.382	34.4	20.4	28.7	44.31	25.34	10.42	1

S5. Estimation of density of broken bonds

The broken bond density on the metal surface is closely affiliated with the surface energy of single crystals and the stepped/kinked nature of the surface.[1,2] The broken bond density per atom (d_{bb}) depends on the crystal orientation, and is geometrically determined for fcc metals as:

$$d_{bb} = \frac{(8h + 4k)}{(h^2 + k^2 + l^2)^{1/2}}$$

where *h*, *k*, *l* denotes Miller indices.

S6. Results of SECCM scan under Argon

Results of the SECCM scan under Ar are presented Figure S2. The trend of lower current density on the secondary facets within the triangle is clear and different from the observation under CO_2 discussed in Figure 3. However, it should be noted that the grains closest to the (100) and (111) poles in the HER scan in Figure S2 are outside the 10° acceptable range of low-index approximation and possess *hkl* parameters of stepped surfaces (see highlighted line 1-3 of Table S1-B), and therefore they cannot be directly compared grain-to-grain to the low-index grains discussed in Figure 2 of the main text. A trend of (110) > (111) > (100) is expected for HER on the primary orientations, but the closest grains to (111) and (110) in Figure S2C are oriented 11.3 and 13.7 degrees away and may explain why the current densities observed on these grains are lower than measurement on the grain that is 6.5 degrees away from (100). Therefore, we have not interpreted them as a trend of HER among the primary orientations. Rather, we have only compared the trend across the entire triangle to deductions of the eCO2RR scan.

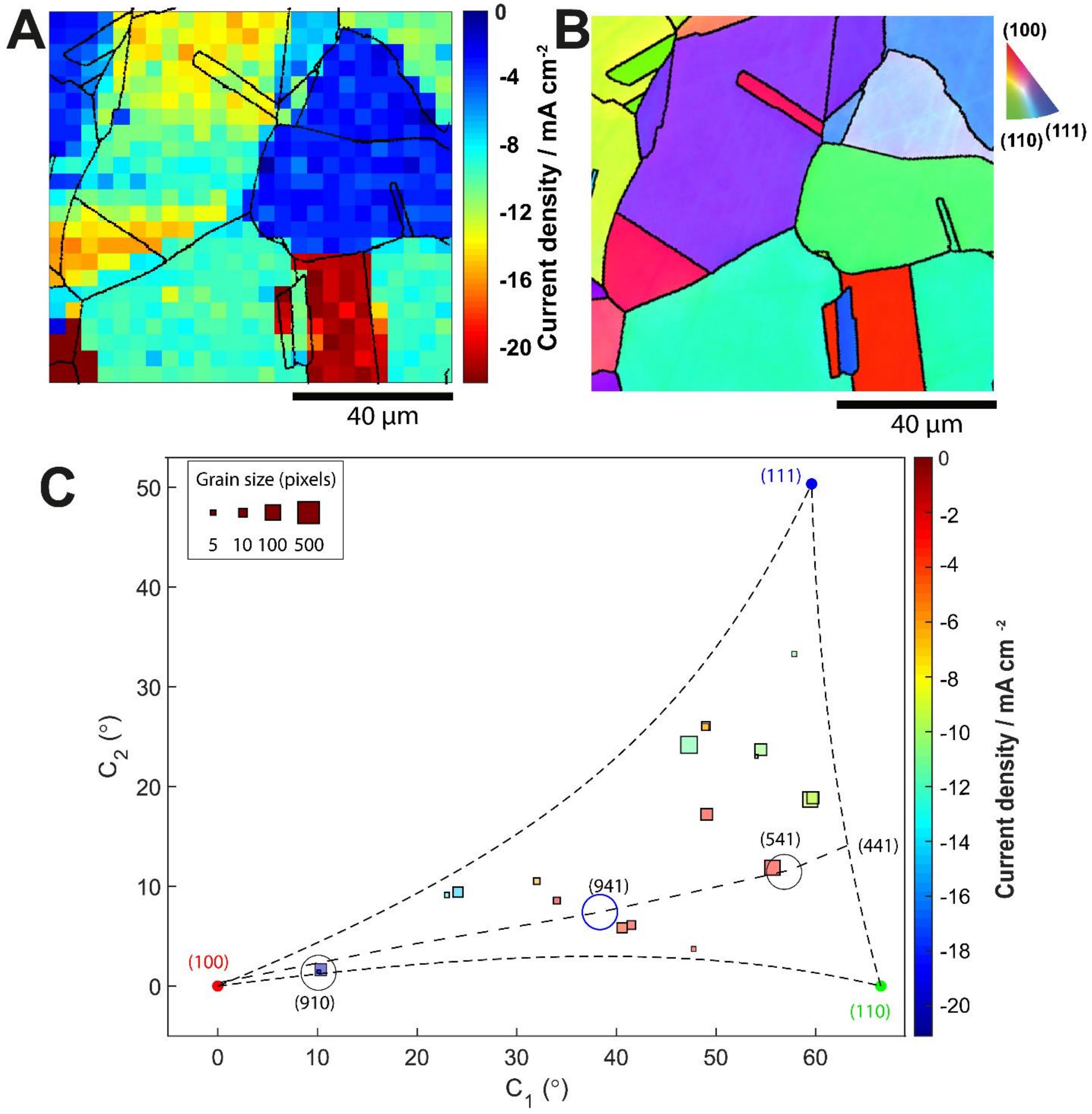

Figure S2: Results of SECCM scan on polycrystalline copper in Ar. (A) Electrochemical image extracted from potentiodynamic SECCM movie (Movie S2) at E_{surf} = -1.05 V vs. Ag/AgCl with an overlay of grain boundaries (black solid lines) from (B) co-located EBSD map. Scan is comprised of 625 pixels. SECCM mapping was conducted with a nanopipet filled with 10 mM $KHCO_3$ in an Ar-purged environmental cell. (C) Corresponding 2-D projection of Cu grain orientations (FCC cubic crystal system) correlated with electrochemical data from SECCM. Dashed lines at the boundaries and bisecting path across the triangle

cover various high index crystallographic structures and are transferred from Figure 3 of the main text for comparison.

S7. XPS results

We investigated the surface composition of as-prepared polycrystalline Cu through ex-situ XPS analysis. XPS measurements were made before and after macroscale electrochemical reduction treatment. Peak fitting of the O1s and Cu $2p_{1/3}$ spectra are presented in SI, Section S7, Figures S3 and S4, respectively. Details of the peaks for Cu oxide materials are in SI (Section S7, Tables S2, and S3). In essence, the results confirmed the presence of Cu_2O (4.57%), $Cu(OH)_2$ (12.10%), and CuO (49.6%) on the as-prepared Cu. After the electrochemical treatment at -1 V vs Ag/AgCl for 1 hour, the values decreased to 0.36%, 0.80%, and 10.89%, in the same order. The removal of surface oxides by electrochemical treatment was also evident in the Cu 2p spectra (SI, Figure S4 of the SI). However, being ex-situ, the XPS signature associated with the oxide features (especially the Cu_2O) was not entirely eliminated due to some reoxidation during sample transfer from electrochemical cell to XPS, for which Cu_2O formation is most rapid. [3]

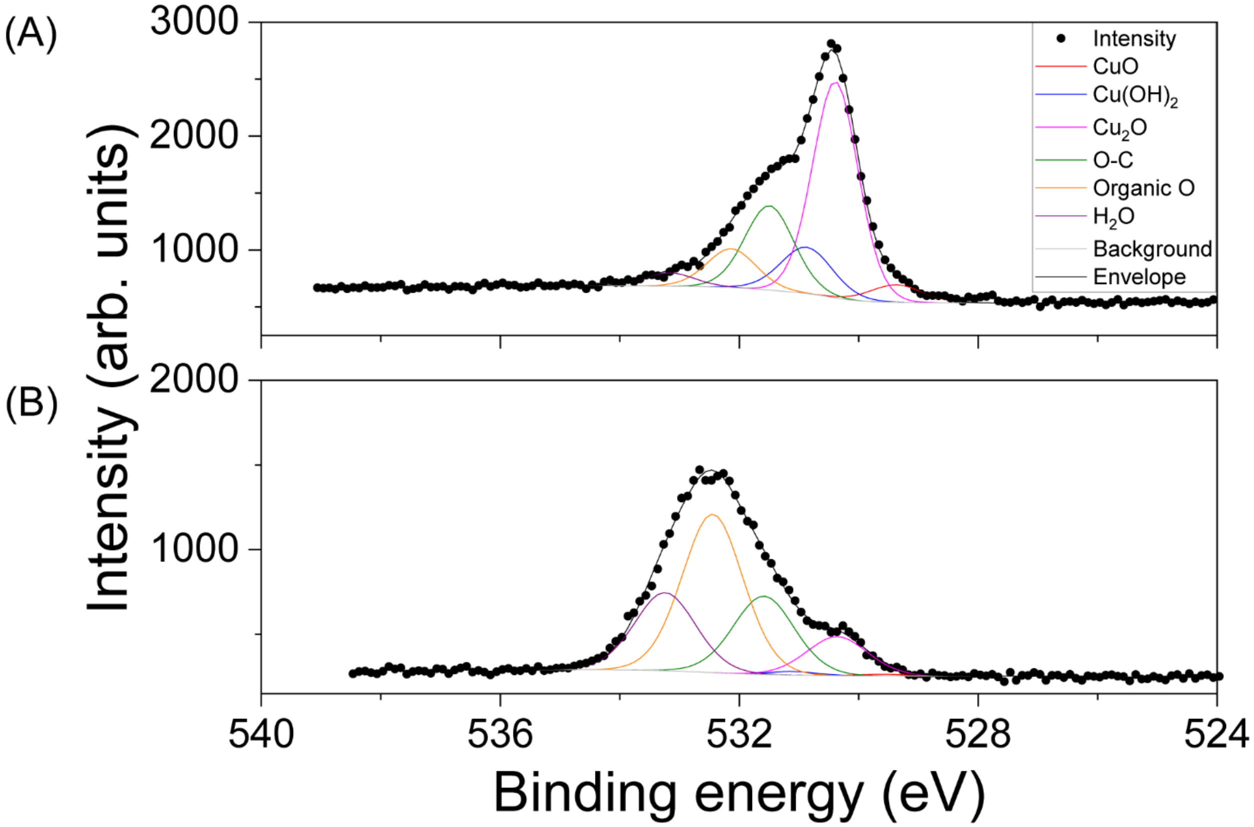

Figure S3: Peak fitting of O1s XPS spectra for polycrystalline Cu surface (A) before, and (B) after treatment at -1.0V *vs.* Ag/AgCl for 1 hour in a three-electrode macroscale setup. (See SI, Section S1).

Table S2: Summary of the O1s peak fitting spectra measured before and after the polycrystalline Cu surface is reduced at -1.0V *vs.* Ag/AgCl in 10mM $KHCO_3$.

**Sample/ treatment detail**	**Material**	**Binding energy (eV)**	**% region counts**
**Before reduction**	CuO	529.37	04.57
	$Cu(OH)_2$	530.87	12.10
	Cu_2O	530.39	49.64
**After reduction**	CuO	529.60	00.36
	$Cu(OH)_2$	531.10	00.80
	Cu_2O	530.36	10.89

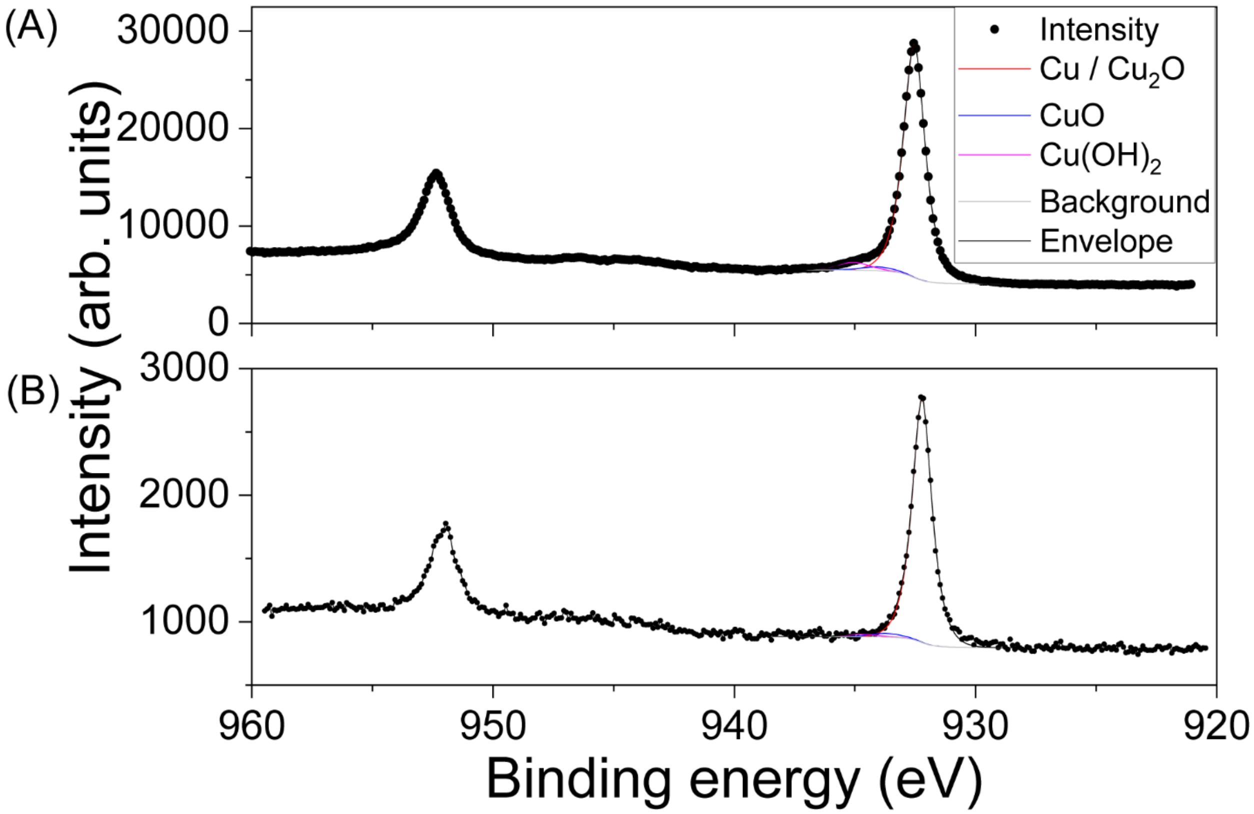

Figure S4: Peak fitting of Cu2p XPS spectra for polycrystalline Cu surface (A) before, and (B) after treatment at -1.0V *vs.* Ag/AgCl for 1 hour in a three-electrode macroscale setup. (See SI, Section S1).

Table S3: Summary of the Cu 2p peak fitting spectra measured before and after the polycrystalline Cu surface is reduced at -1.0V *vs.* Ag/AgCl in 10mM $KHCO_3$

**Sample/ Treatment detail**	**Material**	**Binding *energy* (eV)**	**% region counts**
**Before reduction**	Cu + Cu_2O	932.54	93.76
	CuO	934.04	02.15
	$Cu(OH)_2$	935.06	04.17
**After reduction**	Cu + Cu_2O	932.22	96.34
	CuO	933.71	02.06
	$Cu(OH)_2$	934.74	01.51

S8. References

(1) Hoshi, N.; Hori, Y. Electrochemical Reduction of Carbon Dioxide at a Series of Platinum Single Crystal Electrodes. *Electrochim. Acta* **2000**, *45* (25–26), 4263–4270.

(2) Lang, B.; Joyner, R. W.; Somorjai, G. A. Low Energy Electron Diffraction Studies of High Index Crystal Surfaces of Platinum. *Surf. Sci.* **1972**, *30* (2), 440–453.

(3) Iijima, G.; Inomata, T.; Yamaguchi, H.; Ito, M.; Masuda, H. Role of a Hydroxide Layer on Cu Electrodes in Electrochemical CO_2 Reduction. *ACS Catal.* **2019**, *9* (7), 6305–6319.
